# Surface hardness and flexural strength of dual-cured bulk-fill restorative materials after solvent storage

**DOI:** 10.1186/s12903-023-03047-2

**Published:** 2023-05-19

**Authors:** Bashayer Alzahrani, Abdulrahman Alshabib, Wedad Awliya

**Affiliations:** Department of Restorative Dentistry, College of Dentistry, King Saud University, Riyadh, Saudi Arabia

**Keywords:** Dual-cured, Bulk-fill resin, Surface hardness, Flexural strength, Solvent storage

## Abstract

**Background:**

This study aimed to evaluate the surface hardness (VHN) and biaxial flexural strength (BFS) of dual-cured bulk-fill restorative materials after solvent storage.

**Methods:**

Two dual-cured bulk-fill composites (Surefil One® and Activa™ Bioactive), a light-cured bulk-fill composite (Filtek One Bulk-Fill) and a resin-modified glass ionomer (Fuji II LC) were investigated. Surefil One and Activa were used in the dual-cure mode, all materials were handled according to manufacturer’s instructions. For VHN determination, 12 specimens were prepared from each material and measured after 1 h (baseline), 1 d, 7 d and 30 d of storage in either water or 75% ethanol–water. For BFS test, 120 specimens were prepared (*n* = 30/material) and stored in water for either 1, 7 or 30 d before testing. Repeated measures MANOVA, two-way and one-way ANOVA followed by the Tukey post hoc test (p ≤ 0.05) were used to analyze the data.

**Results:**

Filtek One had the highest VHN, while Activa had the lowest. All materials exhibited a significant increase in VHN after 1d of storage in water, except for Surefil One. After 30 d of storage, VHN increased significantly in water except for Activa, while ethanol storage caused a significant time-dependent reduction in all tested materials (*p* ≤ 0.05). Filtek One showed the highest BFS values (*p* ≤ 0.05). All the materials, except for Fuji II LC, exhibited no significant differences between 1 and 30 d BFS measurements (*p* > 0.05).

**Conclusions:**

Dual-cured materials had significantly lower VHN and BFS compared to the light-cured bulk-fill material. The low results of Activa VHN and Surefil One BFS, indicate that these materials should not be recommended in posterior stress-bearing areas.

## Background

The unique nature of the oral environment challenges the success of resin-based composite (RBC) restorative materials. Dental restorations are subjected to different forces during mastication alongside continuous exposure to oral fluids and fluctuation in temperature. These factors violate the integrity of RBC, resulting in substantial changes as a function of fatigue and aging [1].

Different solvents, especially water, can alter the chemical composition of RBCs, compromising the physical stability and mechanical properties and affecting the integrity of the material’s surface [2]. It has been proved that long-term storage in water has a plasticizing effect on most dental composites due to the destruction of filler–matrix bonds [3] Solvent diffusion is accompanied by a loss in non-reacted components, leading to further degradation of the composite resin material [4]. Ethanol on the other hand is a relevant food-simulating solvent and can be used to simulate accelerated aging. Ethanol has been shown to have a more profound softening effect on resin composites when compared to water [2, 5].The nature of the resin–composite network is also an important aspect that impacts the longevity of the restoration. The chemical composition, microstructure, degree of conversion and crosslinking, along with the matrix–filler interface quality determine the extent of the degradation of restorations [1, 6].

Inadequate polymerization of resin composites is associated with chemical instability, degradation, poor mechanical properties, recurrent caries thus premature failure of restorations [7, 8]. Therefore, the incremental filling technique is recommended to ensure efficient polymerization throughout the material [9, 10]. This technique can be time-consuming and may lead to the introduction of restoration voids if not carried out effectively. Bulk-fill composites were introduced to meet the demand for a faster and more simplified placement technique. These are light-cured materials that can be placed in one increment of up to 4–5 mm. However, deep cavities continue to present a challenge in terms of polymerization efficiency in spite of the improvement in light-cured RBCs [11].

To overcome this limitation, manufacturers have introduced dual-cured bulk-fill materials for direct restorations. These composites consist in general of two initiator systems with light-cured and chemical-cured components. The light-cured initiator is responsible for the polymerization of the top layers and initial hardening. Deeper layers of the material, which receive insufficient light irradiation, are polymerized via a slow chemical-cured reaction [12]. Dual-cured composites generally have a higher degree of conversion values when compared to both light-cured bulk-fill and conventional composites, which might indicate better mechanical properties and degradation resistance [13].

Dual-cured bulk-fill materials such as Surefil One® and Activa™ are marketed for use as direct restorations in posterior-stress-bearing areas. According to the manufacturers, these materials require no capping; thus, they are directly subjected to oral fluids and different loads. Surefil One® and Activa™ are described as ion-releasing materials similar to resin-modified glass ionomers such as Fuji II LC [14]. Ion leaching from the material may impact different properties over time, consequently compromising longevity [15]. A variety of RBC properties are affected by degradation, including surface hardness, wear resistance, dimensional stability, color stability and flexural strength [16].

There is an increasing interest in dual-cure RBCs as direct restorations, however, limited data are available investigating the properties of this type of materials. Comprehensive assessment of these materials is essential to provide accurate recommendations for their clinical applications. The aims of this study were (i) to evaluate the Vickers hardness (VHN) and biaxial flexural strength (BFS) of dual-cured materials in comparison with a light-cured counterpart material and a resin-modified glass ionomer material; and (ii) to investigate the effect of solvent storage on VHN and BFS of these materials. The null hypotheses were as follows:

1. There is no significant difference in VHN between tested materials and within each material after storage in different solvents for different time intervals.
2. There is no significant difference in BFS between tested materials and within each material at the different storage times.

## Methods

### Preparation of specimens

Two dual-cured bulk-fill composites (Surefil One® and Activa™ Bioactive) were evaluated and compared to a light-cured bulk-fill (Filtek One Bulk Fill) and a resin-modified glass ionomer (Fuji II LC). The compositions of the materials investigated in this study are described in Table 1.

**Table 1:** Composition of materials investigated according to information provided by manufacturers

Material	Manufacture	Lot number	Resin matrix	Filler type	Filler load wt%
Surefil One (Dual-cured)	Dentsply Sirona; Konstanz, Germany	2109000686	MOPOS, bifunctional acrylate, acrylic acid, BADEP camphorquinone, self-cure initiator, stabilizer	Aluminum-phoshor-strontium-sodium- fluoro-silicate glass, highly dispersed silicon dioxide, ytterbium fluoride	77%
Activa BioActive-Restorative (Dual-cured)	Pulpdent, Watertown, MA, USA	211117	Diurethane modified with hydrogenated polybutadiene, methacrylate monomers, modified polyacrylic acid camphorquinone,self-cure initiator	Bioactive glass, silica, sodium fluoride	56%
Filtek One Bulk- fill (Light-cure)	3 M ESPE, St. Paul, MN, USA	220721A	AUDMA, UDMA, diurethane-DMA, and DDDMA, AFM, camphorquinone	20 nm silica, 4–11 nm zirconia, cluster Zr-silica, 100 nm ytterbium trifluoride	76.5%
Fuji II LC (Dual-cured RMGI)	GC, Tokyo, Japan	NE09753	HEMA, polyacrylic acid, UDMA,dimethacrylate, camphorquinone	Alumino-fluoro-silicate glass	58%

*RMGI* resin-modified glass ionomer, *MOPOS* modified polyacids, *BADEP* N,N’-diethyl-1,3-bisacrylamido-propan, *AUDMA* aromatic urethane dimethacrylate, *UDMA* urethane dimethacrylate, *DDDMA* (1, 12-dodecanediol dimethacrylate), *AFM* addition-fragmentation monomer*, HEMA* 2-hydroxyethyl methacrylate

G*Power calculator was used for sample size estimation, a total of a hundred sixty-eight specimens was deemed sufficient at a 0.05 level of significance with effect size 0.40 and 90% power. The specimens were prepared using a disc-shaped Teflon mold with an internal 10 mm diameter and 2 mm thickness. A clear Mylar strip was placed over a 1 mm thick glass slide, and the mold was positioned over the strip. The materials were dispensed in the mold and overfilled slightly. Another mylar strip and a glass slide were placed on the top and pressed down firmly. Specimens were cured for 20 s using a high-power (1200 mW/cm^2^) LED-light-curing unit (Bluephase-G2, Ivoclar Vivadent, Schaan, Liechtenstein, Switzerland) that was positioned to be in contact with the glass slide during curing. A calibrated radiometer (MARC™ Resin Calibrator, Blue-light Analytics Inc., Halifax, NS, Canada) was used to verify the irradiance before each use. The specimens were taken from the mold and excess flashes on the edges were removed using a silicon carbide paper with 1000 grit (Buehler, Lake Bluff, IL,USA).

### Surface hardness

Twelve specimens from each material were used for hardness testing. After 1 h of dry storage at 37 ± 1 ^◦^C, baseline surface hardness measurements were recorded using a Vickers Micro-hardness Instrument (FM-700, Kawasaki, Kanagawa, Japan), with a load of 300 g at 23 ± 1 °C for 15 s [5]. Specimens from each material were then divided into one of two groups (*n* = 6) for immersion in different solvents: distilled water or 75% ethanol–water. Top surfaces were marked and the specimens were stored in individual 10 mL glass vials at 37 ± 1 ^◦^C.

**Fig. 1:**
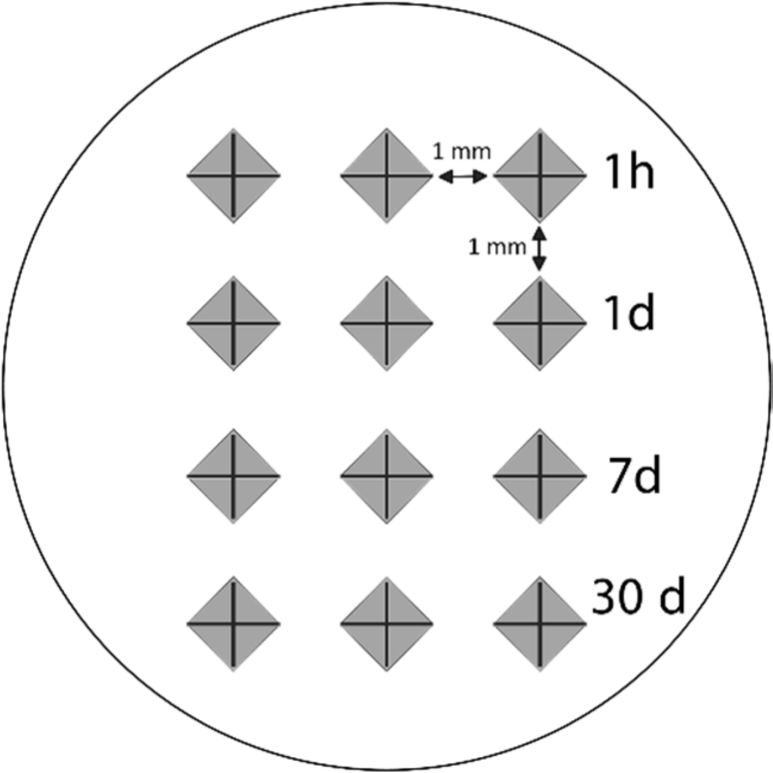
Vickers indentations positioned 1 mm apart vertically and horizontally at the different time intervals

Surface hardness measurements were recorded for each specimen after 1, 7 and 30 d. At each time interval, specimens were removed from the vials and allowed to dry for 1 min before testing. Three indentations were made at each time interval on each specimen as illustrated in Fig. 1. VHN was calculated following this equation:

$$H=1854.4 \frac{P}{{d}^{2}}$$

where H is Vickers hardness in kg/mm2, P is the load in grams and d is the length of the diagonals in µm.

### Biaxial flexural strength test

Thirty specimens were taken from each of the tested materials and divided into three groups (*n* = 10) according to the storage time in water: 1, 7 and 30 d. The specimens were stored in small bottles of distilled water and placed in an incubator at 37 ± 1 ◦C. Specimens were removed from the bottle and allowed to dry for 1 min before testing.

**Fig. 2:**
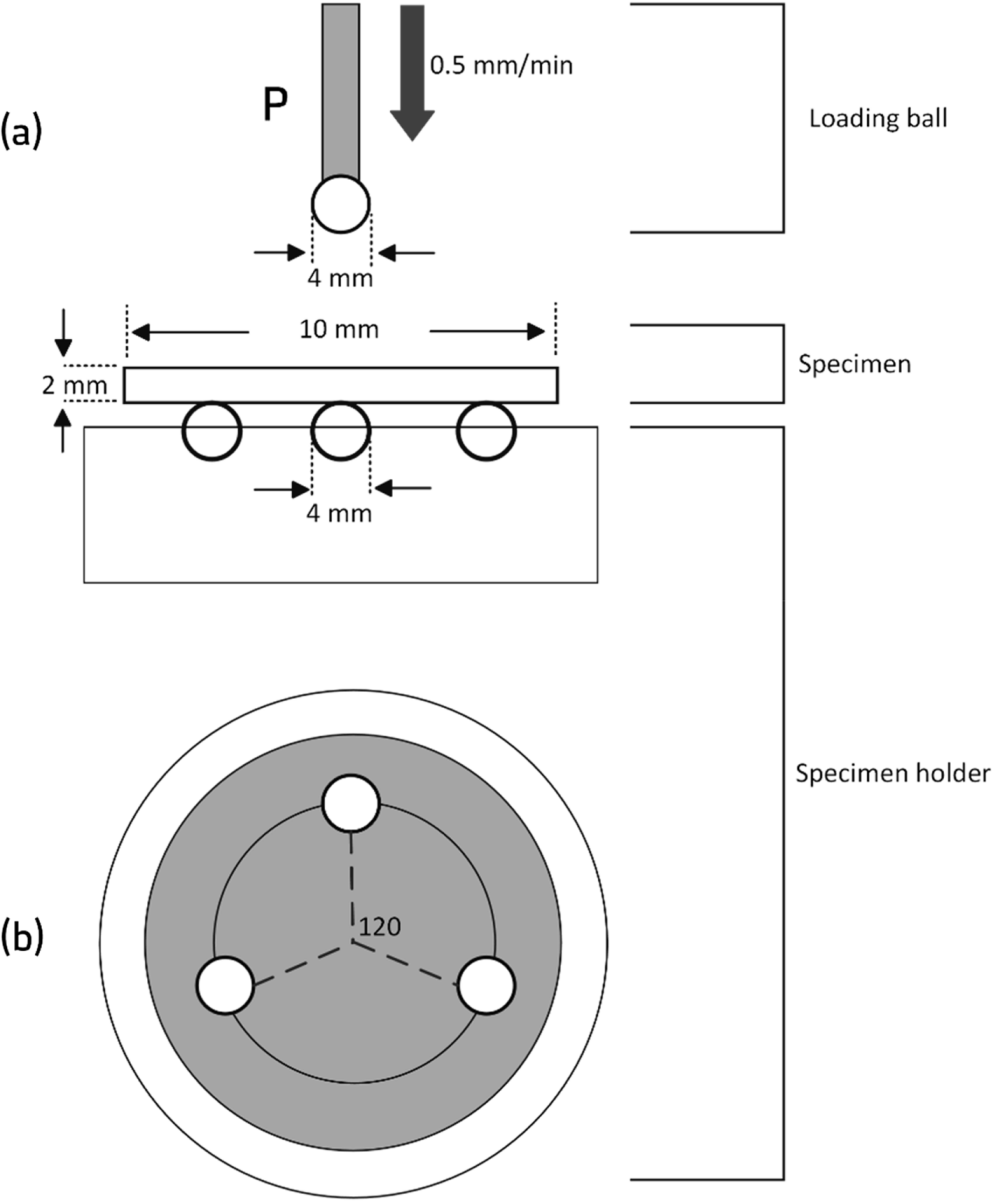
Diagram of the “ball-on-three-ball” biaxial flexural test. (**a)** Frontal view; (**b**) upper view of the disc specimen placed concentrically on 3 balls. P is the loading ball applied at the center of the specimen at a crosshead speed of 0.5 mm/min

A ball-on-three-balls technique in a universal testing machine (Instron- Model 5965, Instron Corp., MA, US) was used for the biaxial flexural test (Fig. 2). Maximum tensile stress value (S), in MPa, was calculated with the following formulas:

$$S=-0.2387P (\mathrm{\rm X}-Y)/{d}^{2}$$

$$\mathrm{X}=\left(1+v\right)\mathrm{ln}{{(r}_{2}/{r}_{3})}^{2}+(\left[1-v\right]/2){{(r}_{2}/{r}_{3})}^{2}$$

$$\mathrm{Y}=\left(1+v\right)(1+\mathrm{ln}{\left[{r}_{1}/{r}_{3}\right]}^{2})+(1-v){{(r}_{1}/{r}_{3})}^{2}$$

where P is the fracture load (in Newtons); d is the specimen thickness (mm); v is the Poisson’s ratio (0.24); r_1_ is the radius of the supporting circle (mm); r_2_ is the radius of the loaded area (mm); r_3_ is the specimen radius (mm).

### Scanning electron microscope (SEM)

Half of the fractured specimens in each group were randomly selected and sputter-coated for 1 min with a thin film (10 nm) of gold (Quorum, Q150R ES, UK). The fractured surfaces were then examined using a scanning electron microscope (JEOL JSM-6610LV SEM, Tokyo, Japan) at 100 × and 350 × magnification.

### Statistical analysis

Data were analyzed using the SPSS version 26.0 software (IBM Inc., Armonk, NY, USA). Descriptive statistics (mean and standard deviation) were used to describe the quantitative outcome variables VHN and BFS. Repeated measures MANOVA followed by one-way ANOVA and Tukey’s multiple comparison test was used to compare the mean values of surface hardness in relation to the study variables: material, solvent across different time points (1 h, 1, 7 and 30 d). Quadratic regression analysis was performed to investigate the relationship between hardness and filler loading. For BFS, two-way ANOVA followed by one-way ANOVA and Tukey’s multiple comparison test was used to compare BFS mean values among the tested materials and storage intervals (1, 7 and 30 d). A *p*-value of ≤ 0.05 was used to report the statistical significance of results.

## Results

### Surface hardness

The VHN data for each of the tested material according to different storage intervals are summarized in Table 2. The interaction between materials, solvents and storage times was statistically significant ( *p* ≤ 0.05). At baseline (1 h dry storage), the comparison of mean values among the tested materials showed statistically significant differences ( *p* ≤ 0.05).

**Table 2:** VHN means and standard deviation (SD) of the tested materials after solvent storage and change % as the difference between 1 h and 30 d

**Material**	**A: Water** **Mean VHN** **(SD)**					**B: 75% Ethanol–Water** **Mean VHN** **(SD)**				
	**1 h**	**1 day**	**7 days**	**30 days**	**Change%**	**1 h**	**1 day**	**7 days**	**30 days**	**Change%**
**Surefil One**	58.4^a,1,2^	59.1^a,1,3^	60.7^a,1,4^	60.9^a,1,5^	** +4.4**	59.2^a,3^	43.3 ^a,1^	42.3 ^a.1^	36.5 ^a,2^	**-38.5**
	(1.8)	(1.3)	(1.4)	(2.7)		(1.1)	(1.0)	(1.1)	(1.0)	
**Activa**	19.0^b,1^	23.8^b,2^	21.6^b,3^	18.9^b,1^	**-0.1**	18.5 ^b,1^	8.5 ^b,2^	7.8 ^b,3^	7.1 ^b,4^	**-61.8**
	(1.2)	(0.9)	(0.6)	(0.62)		(0.6)	(0.9)	(0.3)	(0.7)	
**Filtek One Bulk-fill**	64.9^c,2^	69.8^c,1^	70.2^c,1^	70.5^c,1^	** +8.7**	62.8 ^c,1^	56.9 ^c,2^	55.5 ^c,4^	50.4^c,4^	**-19.8**
	(0.9)	(1.4)	(0.9)	(1.6)		(1.1)	(0.8)	(1.1)	(1.3)	
**Fuji II LC**	45.0^d,2^	48.1^d,1^	55.8^d,3^	46.9^d,1^	**+4.3**	46.5 ^d,1^	38.1 ^d,2^	34.5 ^d,3^	32.2 ^d,4^	**-30.6**
	(1.0)	(0.9)	(1.6)	(1.7)		(1.7)	(0.9)	(1.2)	(1.9)	

For each row, the same superscript number indicates no significant difference (*p* > 0.05)

For each column, the same superscript letter indicates no significant difference (*p* > 0.05)

VHN was significantly influenced by the aging period in both solvents. The highest VHN, both prior to and after storage, was seen in Filtek One, followed by Surefil One, Fuji II LC and finally Activa, which had the lowest VHN among the tested materials.

For the groups stored in water, all specimens exhibited a significant increase in VHN after 1d, except for Surefil One, for which a significant difference was noticed after 7d ( *p* ≤ 0.05). Filtek One demonstrated no significant differences after 1, 7 and 30 days. All materials, except for Activa, showed a significant increase in VHN after 30 d of storage compared to baseline readings (*p* ≤ 0.05). Filtek One showed the highest increase in VHN (8.66%) while Surefil One and Fuji II LC showed comparable increases of (4.42%) and (4.32%), respectively. Activa showed no significant VHN reduction between 1 h and 30 d. For all specimens stored in ethanol, VHN readings after 30 d were significantly lower than baseline at all storage times (*p* ≤ 0.05). All materials showed statistically significant differences between storage intervals, except for Surefil One, which showed no significant difference between mean values at 1 and 7 d. Filtek One demonstrated the lowest VHN reduction (19.7%) while Activa showed the highest reduction (61.7%).

**Fig. 3:**
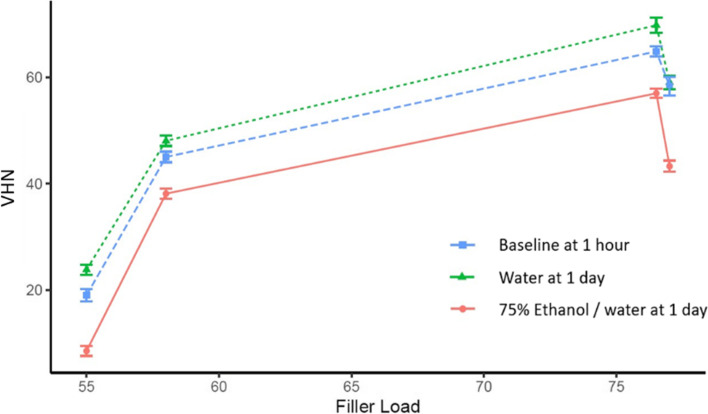
Quadratic regression analysis of filler loading wt% and Vickers hardness (VHN) after baseline (1 h) and 1 d storage in two solvents (water and 75% ethanol–water)

The quadratic regression for the relationship between filler load values and surface hardness shows a highly statistically significant quadratic relation with r^2^ = 0.99 at baseline (1 h dry storage), with r^2^ = 0.97 for storage in water and r^2^ = 0.96 for storage in 75% ethanol (Fig. 3).

### Biaxial flexural strength

The means and standard deviations for the BFS values after different storage times are summarized in Table 3.The mean values of BFS among the tested materials across the storage intervals showed statistically significant differences (*p* ≤ 0.05).

**Table 3:** BFS and standard deviation (SD) of the tested materials measured after storage in water

Materials	1 day	7 days	30 days	Change%
**Surefil One**	25.1^a,1^	32.4^a,2^	29.5^a,1,2^	** + 17.60%**
	(3.5)	(3.8)	(4.6)	
**Activa**	89.4^b,1^	97.9^b,2^	92.3^b,1,2^	** + 3.20%**
	(5.1)	(7.3)	(3.2)	
**Filtek One**	146.3^c,1^	157.9^c,2^	149.3^c,1,2^	** + 2.10%**
**Bulk-fill**	(3.3)	(2.7)	(5.2)	
**Fuji II LC**	29.9^a,d,1^	43.8^d,2^	48.6^d,2,3^	** + 62.30%**
	(3.8)	(4.5)	(4.8)	

For each row, the same superscript number indicates no significant difference (*p* > 0.05)

For each column, the same superscript letter indicates no significant difference (*p* > 0.05)

Filtek One showed the highest BFS values after all storage intervals, followed by Activa. Fuji II LC and Surefil One showed the lowest BFS values with no significant difference at 1 day. All tested materials, except Filtek One, showed significantly higher mean BFS values at 7 d when compared with the mean values at 1 d ( *p* ≤ 0.05). All tested materials, except Fuji II LC demonstrated no significant difference between 1 and 30 d BFS measurements. Fuji II LC showed a significant increase in BFS between 1 and 30 d (*p* ≤ 0.05) and presented the highest change over storage time (62.3%). Surefil One showed a 17.6% increase in BFS, while Filtek One and Activa showed comparable increases in strength (2.1% and 3.2%, respectively).

### SEM examination

**Fig. 4:**
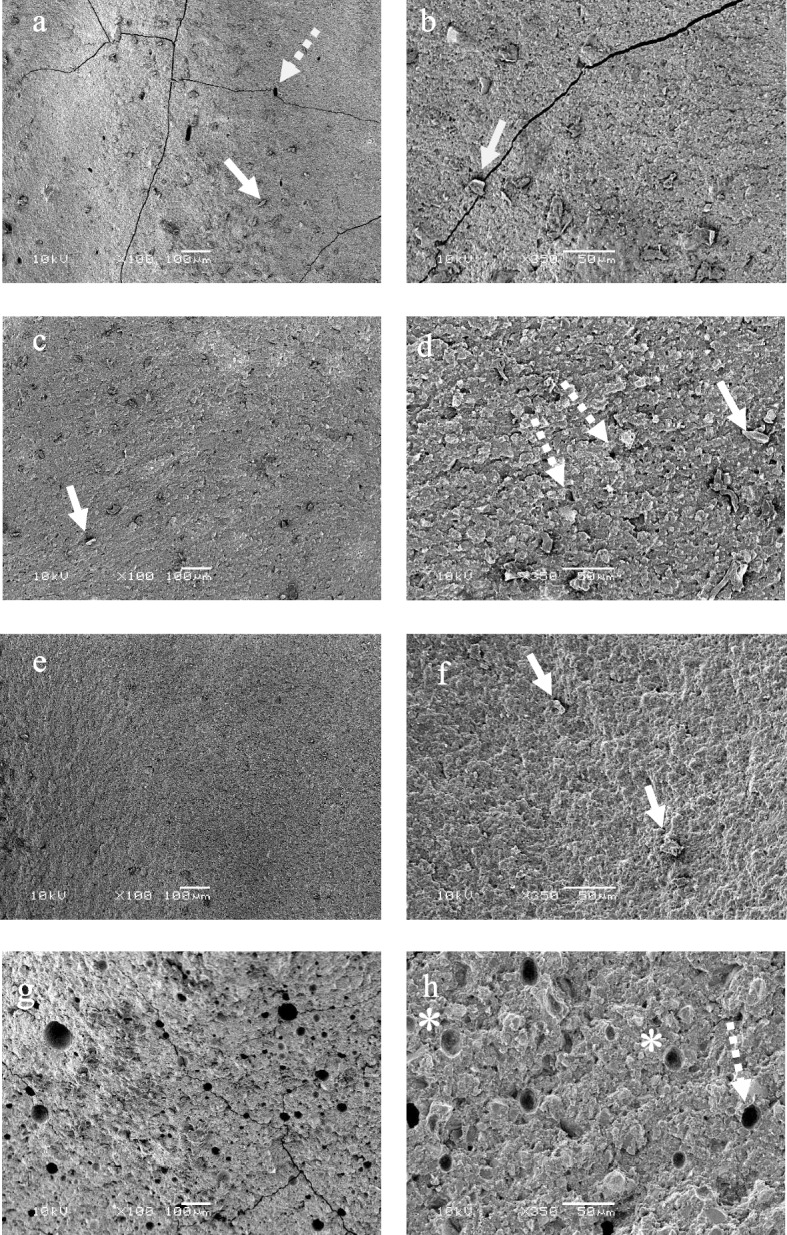
SEM images of Surefil One (**a** and **b**), Activa (**c** and **d**), Filtek One (**e** and **f**) and Fuji II LC (**g** and **h**). The solid arrows indicate exposed filler particles, the dashed arrows denote voids created by particle displacement, and the asterisk indicates a void caused by air entrapment

The SEM micrographs of the fractured surfaces of Surefil One (Fig. 4a and b) clearly showed multiple crack lines and displaced particles. The fractured surfaces of Activa (Fig. 4c and d) showed displaced particles with a few small voids caused by the loss of fillers. Filtek One showed a homogenous surface with few displaced fillers (Fig. 4e and f). Fuji II LC presented craze lines and obvious porosity with multiple voids, either due to filler displacement or void entrapment (Fig. 4g and h).

## Discussion

### Surface hardness

In this study, significant effects on surface hardness were observed according to materials, storage medium and time (*p* ≤ 0.05). Thus, the first null hypothesis was rejected. Filtek One showed the highest VHN, followed by Surefil One, Fuji II LC and Activa.

Hardness indicates the resistance of a material to permanent indentation, which is an important parameter to consider when comparing different materials. An adequate surface hardness is one of the main requirements for restorative materials, especially in posterior-stress-bearing areas [17]. Surface hardness of composite restorations is a direct reflection of the curing quality. The process of polymerization has a notable influence on the hardness of materials, and it has been observed that hardness tends to increase as the degree of conversion during polymerization increases [18]. In the realm of dental restorations, dual-cured composites have garnered attention due to their superior polymerization efficiency, which is expected to result in enhanced mechanical properties. By combining both light-activated and self-curing mechanisms, these composites ensure thorough polymerization even in areas that may be difficult to reach with light curing alone. This comprehensive polymerization is believed to contribute to improved material performance, particularly in terms of hardness and strength [19]. However, hardness is not an isolated material property. It is interconnected with other key characteristics, including the modulus of elasticity and viscosity [20]. Hence, hardness can serve as an indicator of the material's resistance to deformation and wear. Studies have revealed a strong correlation between low surface hardness and poor wear resistance, which can significantly impact the longevity of dental restorations [21, 22].

Various factors related to the composition of the material have been identified as influential in determining surface hardness, including monomer type and ratio, photoinitiators and the degree of polymer crosslinking [17]. Moreover, surface hardness has been found to be strongly influenced by filler load, size, morphology and distribution [23, 24]. Each of these factors can influence the degree of conversion during polymerization, ultimately affecting the resulting hardness of the material. This could explain the different VHN values recorded among the materials tested in this study.

Several studies confirmed a positive correlation between VHN and filler loading; as the filler load increased, higher hardness values were observed [25, 26]. In this study, a positive correlation was confirmed between filler load and hardness. However, the interpretation of the results when considering a single factor, such as filler load, can be deceptive due to the complex nature of the tested materials.

Filtek One showed the highest VHN despite having a filler load comparable with that of Surefil One, which might be attributed to the nano-sized fillers in Filtek One. This is in accordance with previous studies showing that small-sized filler particles have improved hardness. The mean distance between neighboring particles decreases with small-sized fillers, which in turn increases the number of particles at the surface [20, 27]. Surefil One presents a higher filler load and overall smaller and rounder filler when compared to Fuji II LC which could explain the difference in VHN [28]. One study reported that composites with round particles showed improved hardness and flexural strength properties compared with those containing irregular-shaped particles [29].

Activa had the lowest hardness value at the baseline (1 h) measurement and after storage in both solvents. This result is consistent with recent studies comparing Activa with a conventional resin composite and GIC materials [16, 30, 31]. This could be due to its low filler loading (56%) which is mostly composed of bioactive glass fillers that have been showed to reduce surface hardness in previous studies [32, 33]. A study evaluated different ion-releasing materials including Activa showed a reduction in the mechanical properties after long-term storage [34].

The surface hardness of dental composites may be affected by both solvent absorption and contact time with liquids. Several studies revealed a significant influence of different aging media and aging time on the mechanical properties of RBCs [2, 16]. In this study, the results show an increase in VHN for all the materials after 1 d of storage in distilled water when compared to the baseline hardness. This finding is consistent with previous studies and can be attributed mainly to post-irradiation polymerization as well as ongoing acid–base reaction, or self-cure process in dual-cure materials [35, 36].

For Filtek One and Surefil One, the increase in hardness continued for up to 30 days which might suggests that the continuous crosslinking reaction hinder the plasticizing effect of the absorbed water. These results also indicate that these materials require a certain period of time to achieve their maximum degree of polymerization. On the contrary, Activa showed a reduction in VHN after 30 d of storage which could be related to the solubility and the leaching out of the bioactive glass fillers that are not tightly adhered to the matrix [37, 38].

Fuji II LC required 7 days to achieve its maximum hardness. Kanchanavasita et al. found that when RMGICs were immersed in distilled water, 90% of the equilibrium water uptake occurred within 7 days [39]. This was followed by VHN reduction due to water acting as a plasticizing molecule [4].

Regarding storage in ethanol, a significant reduction in VHN after 1d, which continued for over 30 days, was observed for all tested materials. This finding is in agreement with a previous studies that reported a significant reduction in hardness after 1 d of storage in ethanol [2, 5]. Ethanol causes softening of the resin composite surface as it easily penetrates the resin matrix, generating stress at the matrix–filler interface and thereby increasing diffusion and leaching of fillers [39]. Activa showed the highest reduction in VHN after 30 d of ethanol storage. This might be a reflection of filler-matrix debonding as a result of weakened filler surface due to ion leaching [40]. Filtek One showed the smallest reduction (19.76%) in VHN among all the tested materials after storage in ethanol. This might be attributed to its high filler load as well as its hydrophobic resin matrix compared to the other materials. A study evaluated the hardness of 11 bulk-fill RBCs after ethanol storage reported that filler content is a critical factor to determine the resistance to ethanol softening [41]. The high filler ratio in high-viscosity bulk fills such as Filtek One reduces the amount of resin affected by ethanol. The presence of fillers extends the diffusion path length, thus reducing the diffusion coefficient of ethanol [42].

### Biaxial flexural strength

The degradation of mechanical properties in the oral environment alongside growth and accumulation of cracks results in catastrophic failure of restorations [6]. RBCs must have high strength to withstand repeated chewing forces. In vitro flexural testing has been shown to be an appropriate method for assessing the strength of a restorative material [17].In this study, the BFS of the tested materials was evaluated after storage in water for different time intervals (1, 7 and 30 d). Filtek One showed the highest BFS followed by Activa, Fuji II LC and Surefil One. A significant increase in BFS after 7 d of storage was observed for all the tested materials. Thus, the null hypothesis that there would be no significant difference in BFS between tested materials and within each material at the different storage times was rejected.

Flexural strength can be influenced by matrix type, filler size and load and their salinization. Kim et al. observed a significant influence of the filler rate and morphology on the flexural strength, elastic modulus and microhardness of the composites evaluated [24]. Furthermore, filler size was positively correlated to higher flexural strength in seventeen commercial resin composites [26].

Resin composites should ideally be chemically stable, and their mechanical properties should not exhibit significant deterioration as they age. Ferracane et al. investigated the mechanical properties of experimental composites after 2 years of water exposure. It was concluded that water had little influence on flexural strength and modulus [43].

Filtek One, showed the highest BFS with no significant difference between 1 and 30 d of storage. This result agrees with a previous study where Filtek one showed the highest flexural strength and modulus among different bulk-fill resin composites [44].This was expected due to the high proportion of inorganic nanosized fillers. Tanimoto et al. evaluated the effect of filler size on flexural strength; the results showed a reduction in flexural strength with increasing filler particle size. Finite element analysis showed that stress concentration at the filler–matrix interface increased with increasing filler particle size. Smaller filler sizes increase the filler surface area, which increases the uniformity of stress distribution through the material and decreases the stress concentration at the filler–matrix interface [45].

Surefil One showed the lowest BFS values despite the high filler load. This could be attributed to difference in resin-matrix composition and filler characteristics. Surefil one is described as a self-adhesive ion releasing material. In order to allow for adhesion and ion releasing, this material was formulated with a modified polyacid system that possess hydrophilic properties [46]. In the SEM analysis, Surefil One presented obvious extended cracks which could explain its low BFS. The mechanism of microcrack formation was explained by Soderholm et al. as a result of an increased osmotic pressure at the matrix-filler interface due to filler degradation and ion leaching [47].

Activa demonstrated superior flexural strength compared to Surefil One and Fuji II LC which is in accordance with previous studies [30, 48, 49]. According to the manufacturer report, Activa contains a rubberized resin matrix with energy-absorbing elastomeric components (a blend of diurethane and methacrylates with modified polyacrylic acid). UDMA has a relatively high molecular weight (MW = 470 g/mol) and low viscosity with high flexibility. UDMA polymers had significantly higher rates of conversion and crosslinking, resulting in improved flexural strength [50]. Previous studies have reported a notable bend in Acitva specimens before fracture [16, 51]. This was attributed to its low flexural modulus due to the energy-absorbing property rendering the material more flexible [48, 51]. However, higher distortion is expected in materials with low modulus of elasticity. Occlusal load on flexible restorative materials may cause lateral expansion and effect tooth integrity. Therefore, high modulus is necessary for restorative materials in stress-bearing areas to prevent distortion and marginal failure [52].

Despite low initial BFS, Fuji II LC presented the highest increase after 30d (62.3%). This gradual increase in flexural strength over storage time is in agreement with a previous study [53]. Maturation of RMGICs is a complex phenomenon that occurs over time and involves a variety of mechanisms. This development in strength can be attributed to the ongoing acid–base reaction as well as post-irradiation monomer conversion and crosslinking [14].

Based on the results of this study, both dual-cured composites (Surefil One and Activa) showed lower VHN and BFS when compared to the light-cured bulk-fill material. Surefil One showed a high hardness yet demonstrated the lowest strength which suggests that this material should not be recommended in stress-bearing areas.

Activa on the other hand had the lowest hardness but showed significantly high strength. However, considering the flexibility of this material, it can be recommended for restoring cervical lesions as it can flex during function, which in turn minimize the stresses at the tooth/restoration interface and reduce the chances of failure [54].

Laboratory aging provides an indication of materials’ long-term performance. However, this may not be directly reflected in clinical conditions. In this study, water storage did not lead to a significant reduction in the VHN and BFS of dual-cured bulk-fill materials. The 30-day storage period might not be long enough to elicit deterioration of the mechanical properties. Therefore, further studies including longer aging periods are recommended. Moreover, considering the ion-releasing property of these materials, a study investigating the interaction between ion leaching and mechanical properties is recommended.

## Conclusions

Within the limitations of this study, it was concluded that:

- Surefil One and Activa showed significantly lower VHN and BFS when compared to the light-cured bulk-fill material.
- VHN increased significantly in water storage for all the materials except for Activa, while 75% ethanol storage caused significant time-dependent reduction in all tested materials.
- Water storage showed no significant effect on BFS of the dual-cured bulk-fill materials.
- The low results of Activa VHN and Surefil One BFS, indicate that these materials should not be recommended in posterior stress-bearing areas.

## Data Availability

The datasets supporting the conclusions of this article are included within the article.

## Abbreviations

RBC: Resin-based composite
VHN: Vickers hardness number
BFS: Biaxial flexural strength
RMGI: Resin-modified glass ionomer
BADEP: N,N’-diethyl-1,3-bisacrylamido-propan
AUDMA: Aromatic urethane dimethacrylate
UDMA: Urethane dimethacrylate
DDDMA: 1, 12-Dodecanediol dimethacrylate
HEMA: 2-Hydroxyethyl methacrylate
LED: Light-emitting diode
